# USP10 is a potential mediator for vagus nerve stimulation to alleviate neuroinflammation in ischaemic stroke by inhibiting NF-κB signalling pathway

**DOI:** 10.3389/fimmu.2023.1130697

**Published:** 2023-04-20

**Authors:** Chenchen Xie, Xiang Gao, Gang Liu, Hao Tang, Changqing Li

**Affiliations:** ^1^ Department of Neurology, The Second Affiliated Hospital of Chongqing Medical University, Chongqing, China; ^2^ Department of Neurology, Affiliated Hospital & Clinical Medical College of Chengdu University, Chengdu, China; ^3^ Chongqing Key Laboratory of Neurology, The First Affiliated Hospital of Chongqing Medical University, Chongqing, China; ^4^ Department of Geriatrics, Affiliated Hospital & Clinical Medical College of Chengdu University, Chengdu, China

**Keywords:** vagus nerve stimulation, NF-κB signalling pathway, neuroinflammation, USP10, ischaemic stroke

## Abstract

**Background:**

Vagus nerve stimulation (VNS) has a protective effect on neurological recovery in ischaemic stroke. However, its underlying mechanism remains to be clarified. Ubiquitin-specific protease 10 (USP10), a member of the ubiquitin-specific protease family, has been shown to inhibit the activation of the NF-κB signalling pathway. Therefore, this study investigated whether USP10 plays a key role in the protective effect of VNS against ischemic stroke and explore its mechanism.

**Methods:**

Ischaemic stroke model was constructed by transient middle cerebral artery occlusion (tMCAO) in mice. VNS was performed at 30 min, 24hr, and 48hr after the establishment of tMCAO model. USP10 expression induced by VNS after tMCAO was measured. LV-shUSP10 was used to establish the model with low expression of USP10 by stereotaxic injection technique. The effects of VNS with or without USP10 silencing on neurological deficits, cerebral infarct volume, NF-κB pathway activation, glial cell activation, and release of pro-inflammation cytokines were assessed.

**Results:**

VNS enhanced the expression of USP10 following tMCAO. VNS ameliorated neurological deficits and reduced cerebral infarct volume, but this effect was inhibited by silencing of USP10. Activation of the NF-κB pathway and the expression of inflammatory cytokines induced by tMCAO were suppressed by VNS. Moreover, VNS promoted the pro-to-anti-inflammatory response of microglia and inhibited activation of astrocytes, while silencing of USP10 prevented the neuroprotective and anti-neuroinflammatory effects of VNS.

**Conclusion:**

USP10 is a potential mediator for VNS to alleviate neurological deficits, neuroinflammation, and glial cell activation in ischaemic stroke by inhibiting NF-κB signalling pathway.

## Introduction

Ischaemic stroke is the second leading cause of mortality globally (1). Growing evidence indicates that neuroinflammation holds a key role in the pathogenesis of ischaemic stroke (2). A critical modulator of the inflammatory response is the transcription factor nuclear factor-κB (NF-κB) (3), which refers to the complete p50/p65 heterodimeric complex and is bound to the inhibitor of κB (IkBα) protein in the resting state. The activation of the IKK complex can induce the degradation of the IKBα protein which leads to the activation of NF-κB. The IKK complex consists of three subunits: IKKα, IKKβ and the essential regulatory subunit NF-kappa-B (NEMO, also known as IKK-γ). NEMO has a ubiquitin-binding structural domain that binds to polyubiquitin chains. The polyubiquitination of NEMO is essential for IKK activation (4). Injury can cause polyubiquitination of NEMO, which resulted in the dissociation and translocation of p65 from the cytoplasm to the nucleus (5). NF-κB activation has been shown to be indispensable for the transcriptional induction of pro-inflammatory mediators involved in immunity, such as cell adhesion molecules and cytokines (6). Inhibition of NF-κB activation can reduce cerebral injury and inflammation induced by ischaemic stroke (7).

Previous studies have well demonstrated the involvement of vagal nerve activity in regulating inflammation (8). The “inflammatory reflex” describes a neural circuit that is capable of transmitting information about the inflammatory state of the body to the brain and regulating immunity through signaling by the vagus nerve (9). Bidirectional vagus nerve signaling is the major component of this immunomodulatory circuit. The most effective therapeutic approaches for ischaemic stroke are intravenous thrombolysis and endovascular therapies which must be applied within a precise time window (10). The current time window for intravenous thrombolysis is within 4.5 hours of ischemic stroke onset, while that for endovascular treatment should be no more than 24 hours. In this second case, the exact time window needs to be determined on the basis of imageological diagnosis (10). Unfortunately, most ischaemic stroke patients fail to receive these treatments within the appropriate time window. Therefore, this limitation makes other complementary treatment approaches particularly important. Vagus nerve stimulation (VNS), as one of the brain stimulation modalities with a history of over 150 years, was first approved by the FDA as an alternative therapy for refractory epilepsy (11) and is now recognized as an effective treatment for ischaemic stroke (12). In animal models with cerebral ischemia/reperfusion, VNS had a neuroprotective effect by reducing infarct volume and neurological deficits, partly mediated by its anti-inflammatory properties (13). Our previous findings indeed showed that VNS exerted its neuroprotective effect via the suppression of apoptosis and pyroptosis, as well as through the modulation of neuroinflammation (14, 15). Furthermore, VNS was shown to suppress microglia activation and reduce neuroinflammation by regulating the NF-κB pathway (16). However, the exact mechanisms underlying this modulation are poorly characterized and require further exploration.

Ubiquitin-specific protease 10 (USP10), a member of the ubiquitin-specific protease family, is ubiquitously expressed in many cell types, including neurons (17). USP10 regulates key inflammation-related signals, cell proliferation, apoptosis, autophagy, and cancer metabolism (18). USP10 can inhibit the activation of NF-κB and the release of inflammatory cytokines (5, 19) while USP10 silencing can markedly increase NF-κB activation (5, 19). Wang et al. discovered that USP10 may play a protective role against ischemic stroke by suppressing inflammation, apoptosis, and JNK activation via direct interaction with transforming growth factor-β activated kinase-1 (TAK1) (20).

Our study found that USP10 expression in the brain was decreased after ischaemic stroke, and VNS treatment could counteract it by promoting USP10 expression. However, the studies related to the regulation of USP10 after ischemic stroke are limited, and the specific mechanisms underlying how USP10 exerts its role in ischemic stroke need to be elucidated. Based on these findings, we hypothesized that VNS achieves cerebral protection by influencing NF-kB-related neuroinflammation via USP10 regulation. In the present study, we clarified the protective effects of VNS following ischaemic stroke and explored the underlying mechanisms. Our results revealed that the VNS-induced enhancement of USP10 was a mechanism to attenuate neurological dysfunction, neuroinflammation, and glial cell activation caused by ischaemic stroke, and this effect was achieved by the modulation of the NF-κB signaling pathway.

## Materials and methods

### Animals

A total of 200 mice (20-25 g) were purchased from the Experimental Animal Center of Chongqing Medical University. Mice were maintained in a 12/12h light/dark cycle with free access to food and drink at the temperature and relative humidity of 21-23°C and 60%, respectively. All procedures were conducted in accordance with the National Institutes of Health’s Guidelines for Animal Care and Use. All animal procedures were authorized by animal experimental ethical inspection of the Second Affiliated Hospital of Chongqing Medical University (No. 2022kls119).

### Construction of the transient middle cerebral artery occlusion model

The tMCAO model was established using the previously described method (21, 22). Briefly, the mice were anesthetized intraperitoneally with 3.5% chloral hydrate (0.1ml/10g). A midline neck incision was performed to expose the right common carotid artery (CCA), external carotid artery (ECA), and internal carotid artery (ICA). The origin of middle cerebral artery (MCA) was blocked for 60 mins by inserting a nylon suture through the carotid bifurcation. Then, the suture was gently removed for reperfusion. In the sham group, similar surgical procedures were performed except for suture insertion at the origin of MCA. Throughout the operation, a thermostatically controlled infrared lamp was applied to keep the body temperature at 37 °C. The 200 mice were randomly divided into the following groups: Sham group (23), Sham+VNS group (10), tMCAO group (24), tMCAO+VNS (24), tMCAO+VNS+LV-Scramble (24), tMCAO+VNS+LV-shUSP10 (24). The Sham and Sham+VNS groups were used to explore the effects of VNS on sham surgery mice. Mice in the Sham+VNS group only received electrical stimulation without any occlusion of the CCA and MCA. The tMCAO model was successfully established if the regional cerebral blood flow decreased to 20% after occlusion and returned to more than 80% of baseline after reperfusion, as measured by a laser Doppler flowmeter (PeriFlux 5000, Perimed AB, Sweden). Meanwhile, mice were evaluated by Longa scoring (21) for neurological defects immediately after resuscitation, and those scoring 2 or 3 were enrolled in the study and the mice that did not meet the required score were excluded from the study (25). The number of deaths in each group at 72h after tMCAO: sham (0), tMCAO (4), tMCAO+VNS (2), tMCAO+VNS+LV-scramble (2), and tMCAO+VNS+LV-shUSP10 (3).

### *In vivo* lentivirus injection

**Figure 1:**
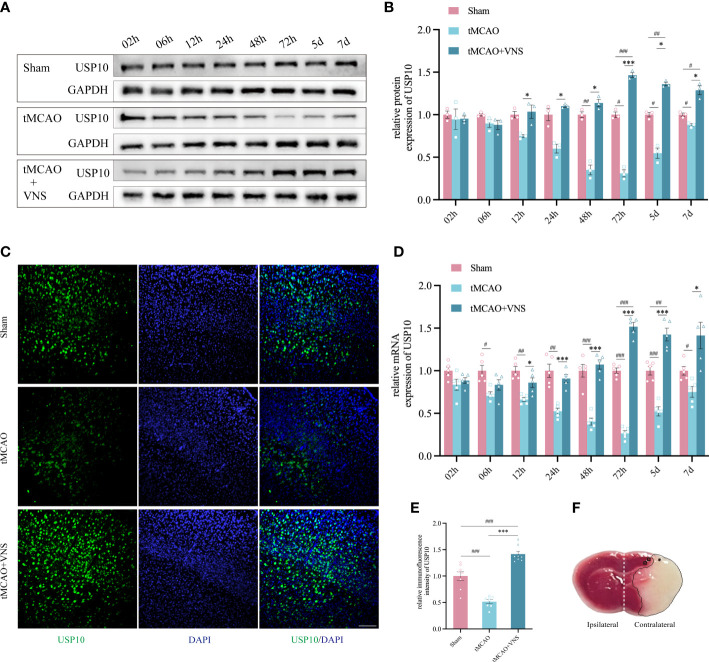
VNS enhanced USP10 expression in mice with ischaemic stroke. **(A)** Western blotting results of USP10 protein levels in each group. **(B)** Histogram of the USP10 protein expression. n=3. **(C)** The USP10 expression in the cortex at 72 hr was examined by immunofluorescence. Scale bar=100 μm. **(D)** The RT-qPCR results of USP10 mRNA levels at each time point. n=5. **(E)** Histogram showed the relative immunofluorescence intensity of USP10 (n=8). **(F)** Relevant areas after tMCAO shown by TTC staining of coronal brain sections: * represents the core area of ischemia; □ represents the ischemic penumbra of the cerebral cortex; the ipsilateral and contralateral cerebral hemispheres are shown with white dashed lines. Data are mean ± SEM. ***P<0.001, and *P<0.05 vs. the Sham group; ###P<0.001, ##P<0.01, and #P<0.05 vs. the tMCAO group.

Two weeks before tMCAO, mice were anesthetized and mounted in a stereotaxic apparatus (Stoelting, USA). Lentivirus contained USP10 shRNA was constructed by Genechem (Shanghai, China) using hU6-MCS-CBh-gcGFP-IRES-puromycin. USP10shRNA sequence: sense: 5′-ccggCACCTGAAGCGCTTCGTCTATctcgagATAGACGAAGCGCTTCAGGTGtttttg-3′;anti-sense:aattcaaaaaCACCTGAAGCGCTTCGTCTATctcgagATAGACGAAGCGCTTCAGGTG-3′. Lentivirus including shUSP (1×10^9^ TU/ml) and Scramble (2×10^9^ TU/ml) were injected according to the following microinjection coordinate (injection sites are depicted in **Figure 1A**): AP + 0.5 mm; L -2.0 mm; V -3.0 mm(cortex) and AP + 0.5 mm; L -2.0 mm; V -1.5 mm(striatum) (26, 27). The purpose of injecting the striatum is to maximize the transduced brain area and suppression USP10. A 10µl Hamilton syringe was used to complete the injection at a rate of 0.2 µl/min. After 10 mins, the needle was withdrawn, and the wound was closed with a suture.

### VNS treatment

VNS was performed firstly at 30 mins, 1 day, and 2 days after the establishment of tMCAO. Invasive VNS was performed as previously described (28). Briefly, the mice were anesthetized intraperitoneally with 3.5% chloral hydrate (0.1ml/10g). Bipolar electrodes were wrapped delicately around the right cervical VN and sutured to the sternocleidomastoid muscle. VNS (0.5 mA, 5 Hz) was administered through a Grass Model S48 stimulator (Grass Technologies, USA) for 30 seconds every five minutes for one hour.

### Neurobehavioural assessment

The neurobehavioral deficits were evaluated by Longa’s score, the modified Neurological Severity Score (mNSS) and the rotarod test at 72hr after tMCAO by an investigator who was blinded to the trial. The neurobehavioral tests were conducted in a dedicated room with no natural light, noise, odor, or other animals not involved with testing. Longa’s score was performed as described previously. The mNSS evaluates motor, sensory, reflex, and balance ability, rating from 0 to 18, with higher ratings indicating a more severe neurological deficit (29).

The rotarod test was performed as previously described (30). Mice were placed on a drum rotating at the speed increasing from 4 rpm to 40 rpm in 5 mins. The time when the mouse fell off the drum was recorded and labeled as latency to fall. Prior to tMCAO, each mouse underwent five trials, with the mean latency to fall in the third, fourth, and fifth trials serving as the baseline. At 72hr following tMCAO, mice underwent another 5 trials with a minimum 5-min gap between each session, with the mean latency to fall calculated with the data from the third through fifth trials. The results of the rotarod test were reported as a percentage of the mean latency after tMCAO divided by the mean latency before tMCAO, with lower scores indicating more severe neurological deficits.

### TTC staining

2,3,5-triphenyltetrazolium chloride (TTC, Sigma-Aldrich, USA) staining was performed to detect cerebral infarct volumes at 72hr after tMCAO (25). The mice were euthanized, and the brains were rapidly removed and frozen at -20°C for 20 mins. Five 2-mm-thick coronal slices of the brain were prepared and stained with 2% TTC for 25 mins at 37.5°C, followed by immersion in 4% paraformaldehyde for 12 hrs. The stained slices were photographed and analyzed in Image J. The infarct volume was determined as follows: percentage hemisphere lesion volume = (total infarct volume - (right hemisphere volume - left hemisphere volume))/left hemisphere volume×100%.

### Western blot

Mice were euthanized at 72hr after tMCAO, and the ischemic penumbra of the cerebral cortex was quickly obtained. Protein samples were extracted by lysing the tissue using RIPA lysis buffer supplemented with PMSF and phosphatase inhibitors (Beyotime, Shanghai, China). In addition, nuclear and cytoplasmic proteins were extracted with the use of the Nuclear and Cytoplasmic Protein Extraction Kit (No. P0027, Beyotime, Shanghai, China). The concentrations of proteins were determined with the BCA kit (Beyotime, Shanghai, China). Western blot was performed as described previously. PVDF membranes were incubated overnight at 4°C with primary antibodies: anti-USP10 monoclonal rabbit antibody (no. 8501S, Cell Signaling Technology, USA, 1:1000), anti-β-actin rabbit antibody (no. 4970, Cell Signaling Technology, USA, 1:1000), anti-IκBα rabbit antibody (no. 4812, Cell Signaling Technology, USA, 1:1000), anti-NF-κB p65 rabbit antibody (no. 8242, Cell Signaling Technology, USA, 1:1000), anti-phosphorylated IκBα (Ser32) rabbit antibody (no. 2859, Cell Signaling Technology, USA, 1:500), anti-Lamin B rabbit antibody (No. 17416, Cell Signaling Technology, USA, 1:1000). The membranes were then treated for an hour at 37°C with the respective secondary antibodies. The images were captured by the Fusion FX5 Analysis System. The intensity of each protein band was analyzed blindly using Quantity One software and compared with that of the GAPDH or β-actin protein band. The quantitative data obtained for the target proteins were normalized to the sham control group for statistical analysis and graphical presentation.

### Immunoprecipitation

Immunoprecipitation with NEMO antibody was performed after dissociation of samples with IP lysis buffer plus 1% SDS. Then it was boiled for 5 min at 95 °C. Supernatants were diluted with IP buffer, and NEMO antibody (No.2685, Cell signal technology, USA) was added to perform the immunoprecipitation. In all, the transferred nitrocellulose membrane was further boiled in water for 30 min and then probed with an anti-Linear Ubiquitin Antibody (ZRB2114, Millipore, USA). Finally, the membrane was visualized and analyzed.

### RT−qPCR

Total RNA was isolated using Trizol reagent (Takara Biotechnology, Japan) from the ischaemic penumbra of the cerebral cortex according to the manufacturer’s instructions. cDNA was synthesized using the reverse transcription kit (Takara, Japan). The amplification conditions were as follows: 5 minutes at 95°C; 40 cycles of 10s at 95°C and 30s at 60°C. Each amplification was conducted three times with different RT reactions. In the same sample, the expression of GAPDH was used to normalize the expression of each targeted gene. The 2^−ΔΔ^Ct technique was utilized to determine relative gene expression. The primers are listed in **Table 1**.

**Table 1:** List of primers for RT-qPCR.

Gene	Sequence	
USP10	Forward	5’-CTGAAGCCGTTGAAAAAGATGAG-3’
	Reverse	5’-TCAGCCTCTGCGTTAGAGTTG-3’
TNF-α	Forward	5’-TGCTGCAGGACTTGAGAAGA-3’
	Reverse	5’-GAGGAAGGCCTAAGGTCCAC-3’
IL-1β	Forward	5-CCAAAAGATGAAGGGCTGCT-3′
	Reverse	5-ACAGAGGATGGGCTCTTCT-3′
IL-6	Forward	5’-AAAGAGGCACTGGCAGAAAA-3’
	Reverse	5’-TTTCACCAGGCAAGTCTCCT-3’
GAPDH	Forward	5’-GGTTGTCTCCTGCGACTTCA-3’
	Reverse	5’-TGGTCCAGGGTTTCTTACTCC-3’

### Immunofluorescence

Immunofluorescence staining was conducted on 15μm thick frozen brain sections (31). Sections were initially treated with 1% Triton X-100 for 30 mins followed by blocking with 5% bovine serum albumin under 37°C for 1 hr. Then the sections were kept at 4°C overnight for incubation using the following primary antibodies: anti-GFAP mouse antibody (no. 80788, Cell Signaling Technology, USA, 1:300), anti-Iba-1 Chicken antibody (Cat234006, SYSY, Inc., Germany, 1:400), anti-CD68 rabbit antibody (No. 97778, Cell Signaling Technology, USA, 1:400), anti-CD206 rabbit antibody (No. 24595, Cell Signaling Technology, USA, 1:400), anti-USP10 rabbit antibody (no. 8501S, Cell Signaling Technology, USA, 1:300). After that, the sections were incubated with corresponding secondary antibodies: Alexa Fluor 488-conjugated goat anti-mouse IgG (H + L; SA00006-1, Proteintech, 1:300), Alexa Fluor@647-conjugated goat anti-chicken IgY (ab150171, Abcam, 1:400), Alexa Fluor 488-conjugated goat anti-rabbit IgG (bs-0295G-AF488, Bioss, 1:300). DAPI was utilized to achieve the staining of the nuclei.

Images of microglia (Iba-1^+^cells, CD68^+^/Iba-1^+^cells, and CD206^+^/Iba-1^+^cells), astrocytes (GFAP^+^cells), and USP10^+^ cells were taken blindly using an A1+R laser confocal microscope (Nikon, Tokyo, Japan) with a 20x objective. Cell counts were calculated using ImageJ software (NIH, MD, USA) by a blinded investigator. The ratio of total cell body size to cell size and the skeleton analysis of microglia were calculated to assess the morphological changes of microglia. The Z-stack confocal images (60× oil microscope) were acquired with an interval of 0.5 μm. Maximum intensity projection images were converted from Z-stack images using Image Fiji software. The ratio of total cell body size to cell size was performed according to the method described by Hovens et al. (32). Briefly, intensity thresholds and size filters were applied using the adjusted threshold and analysis particle functions. To measure the total cell size, the threshold was kept at the level automatically provided by the program with no size filter applied. For the measurement of total cell body size, the threshold was lowered by 40 points and a size filter of 150 pixels was applied. For the skeleton analysis, the images were converted to binary and skeletonized images (33) to collect data by employing the Analyze Skeleton (2D/3D) plugin.

### Flow cytometry

Mice were anesthetized and perfused with cold Hank’s Balanced Salt Solution (HBSS), then the pre-infarct cerebral cortex was removed and dissected into small pieces. Neural Tissue Dissociation Kit (Trypsin) (Miltenyi Biotech) was used to prepare single-cell suspensions. Single-cell suspensions were filtered through a 70 μm membrane and centrifugated and resuspended in 30% Percoll, covered to the bottom of the solution with 70% Percoll, and microglial-rich cell populations isolated from the 30-70% interval were diluted in ice-cold PBS and recovered by cold centrifugation in a microcentrifuge tube. The obtained cells were incubated with a mixture of antibodies against CD11b-PE (No. 12-0112-82, eBioscience, 1:300) and CD45-Pacific Blue (No. MCD4528, eBioscience,1:300), at 4°C for 30 min, and CD11b^+^ CD45^int^ cells were sorted by Agilent NovoCyte Flow 3000 (USA) as microglia. For flow cytometry analysis, the following antibodies were used: CD68-APC (No. MA5-23616, eBioscience, 1:300), CD206-APC-Cy7 (No. LS-C732921, LSBiO, 1:300). Appropriate isotype controls were used according to the manufacturer’s instructions (ThermoFisher eBioscience) and the results were analyzed using Novoexpress software.

### Enzyme−linked immunosorbent assay

The ischaemic penumbra of the cerebral cortex was taken to determine the levels of interleukin-6 (IL-6), interleukin-1 beta (IL-1β), and tumor necrosis factor-alpha (TNF-α) at 72hr after tMCAO with the use of ELISA kits (no. EK0527, EK0394, and EK0411, BOSTER Co.), as directed by the manufacturer.

### Statistical analysis

All of the data collected were processed randomly and appropriately blocked in this study. The data are presented as mean ± SEM (34). The graphs were generated using GraphPad Prism 8.0. IBM SPSS Statistics 27.0 was used to conduct the statistical analysis. All data with a significant difference were analyzed by GPower 3.1 to obtain effect size and power value. The differences in USP10 mRNA and protein expression between groups were analyzed using two-way ANOVA with Bonferroni *post hoc* tests. The Iba-1^+^ cell counts and the percentages of CD68^+^ + CD206^+/^IBA^+^ and CD206^+^/IBA^+^ cells of immunofluorescence images, as well as the ratio of CD68/CD206 and CD68^+^+ CD206^+/^CD11b^+^CD45^int^ of flow cytometry results, were compared between groups with Dunnett’s T3 tests. The one-way ANOVA was used for multiple comparisons of all other quantitative data if the experimental data were normally distributed and the variance was homogeneous. A statistical significance threshold was determined as P < 0.05.

## Results

### VNS enhanced USP10 expression following ischaemic stroke

To explore the USP10 expressions at different times and the effect of VNS on its expression after ischaemic stroke, the protein and mRNA levels in the ischaemic penumbra of the cerebral cortex were assessed by western blot and RT-qPCR, respectively within 7 days following reperfusion (**Figures 1A, B, D**). Randomly, mice were divided into groups designated as Sham, tMCAO, and tMCAO+VNS. **Figure 1F** shows TTC staining of coronal brain sections from tMCAO mice, demonstrating that the tMCAO modeling method can successfully establish cerebral infarction due to right middle cerebral artery obstruction. In the tMCAO group, the expression of USP10 gradually decreased until to a minimum at 72hr and then gradually increased. USP10 mRNA and protein levels were significantly higher in the tMCAO+VNS group than in the tMCAO group at the respective time points, except at 2hr and 6 hr, (**Figures 1A, B, D**).

The USP10 protein expression in the ischaemic penumbra of the cerebral cortex in each group was further analyzed by immunofluorescence at 72 hr after reperfusion. The immunofluorescence intensity of USP10 positive expression was significantly suppressed in the tMCAO group compared with the Sham group. VNS treatment could obviously increase the expression of USP10, which is similar to the result of RT-qPCR and Western blot (**Figures 1C, E**). These results showed that the upregulation of USP10 exerted by VNS treatment was most significant at 72h. Therefore, in order to explore whether USP10 is the potential mediator for VNS to alleviate neuroinflammation in ischaemic stroke, we chose 72h post-tMCAO as the time point for the follow-up experiments.

### USP10 silencing reversed the protective effects of VNS against ischaemic stroke

**Figure 2:**
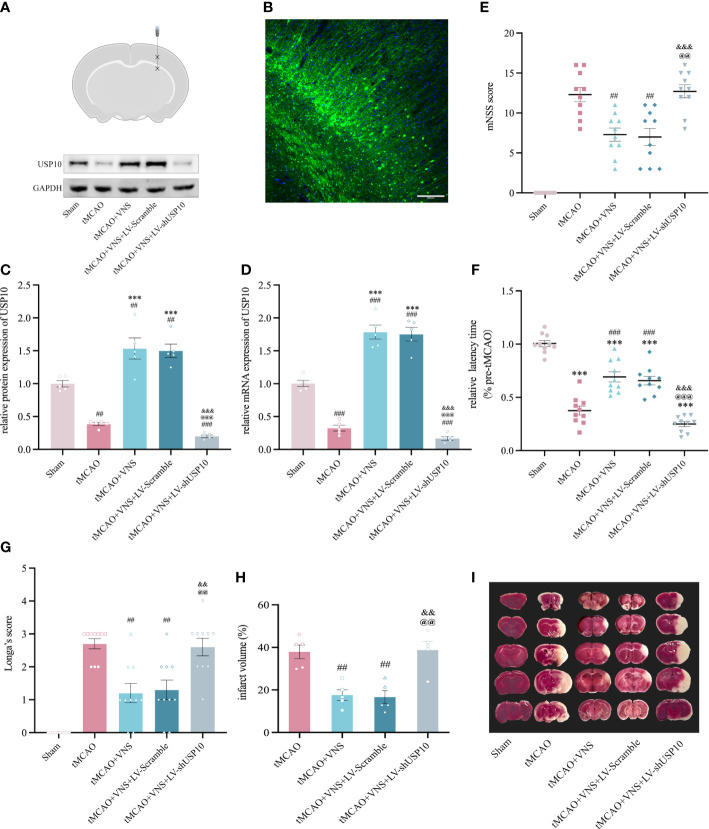
USP10 silencing suppressed the neuroprotective effect of VNS in mice with ischaemic stroke. **(A)** Schematic diagram of a mouse brain coronal section. Asterisk designates sites of lentiviral injection. **(B)** Distribution of lentiviral mediated gene transduction. The GFP fluorescent protein in the vector can be expressed by the construct as a marker. The blue color shows the nuclei stained with DAPI. **(C, D)** USP10 expression in mice receiving LV-shUSP10 was assessed using western blotting (C, n=3) and RT-qPCR (D, n=5). **(E–G)** The neurobehavioral function was evaluated using mNSS Score **(E)**, and rotation test **(F)** Longa Score **(G)**. n=10. **(H)** Quantitative evaluation of brain infarct volume. n=5. **(I)** TTC staining images (white: infarct area; red: non-infarct). Data are mean ± SEM. ***P<0.001 vs. the Sham group; ###P<0.001 and ##P<0.01 vs. the tMCAO group. @@@P<0.001 and @@P<0.01 vs. the tMCAO+VNS group. &&&P<0.001 and &&P<0.01 vs. the tMCAO+VNS+LV-Scramble group.

To explore whether VNS exerts a neuroprotective effect by upregulating USP10 expression, LV-shUSP10 was utilized to silence the USP10 expression in the ischaemic cortex (Figures 2 A, B). Mice were divided into five groups randomly named as: Sham, tMCAO, tMCAO+VNS, tMCAO+VNS+LV-Scramble, and tMCAO+VNS+LV-shUSP10. Levels of USP10 protein and mRNA expression were detected by Western blot and RT-PCR. At 72 hr after reperfusion, USP10 protein and mRNA levels were lower in the tMCAO+VNS+LV-shUSP10 group than in the tMCAO+VNS groups (**Figures 2C, D**), indicating that LV-shUSP10 intervention could significantly inhibit USP10 expression.

Longa’s score, mNSS score, and rotarod test were employed to assess the severity of neurological deficits in mice. As illustrated in **Figures 2E–G**, mice in the tMCAO group exhibited significant neurobehavioral dysfunction, while VNS treatment significantly rescued neurological function in tMCAO mice, as characterized by lower rating in mNSS and longer latency to fall in the rotarod test. However, such improvement was significantly attenuated by the LV-shUSP10 intervention, as demonstrated by the poor neurobehavioral improvement in the tMCAO+VNS+LV-shUSP10 group. Furthermore, to reveal whether USP10 silencing could attenuate the reduction of cerebral infarct volume by VNS, TTC staining was performed. As shown in **Figures 2H, I**, the sham group did not show cerebral infarction; the tMCAO group had a significant cerebral infarction in the right hemisphere. VNS treatment reduced the cerebral infarct volume, while this effect of reduction was inhibited upon USP10 silencing.

To evaluate whether VNS affects the neurological function score, cerebral USP10 expression, and neuroinflammation in the Sham surgery group. Mice were randomly divided into Sham and Sham+VNS groups. We found that both the Sham group and Sham+VNS group scored 0 on the Longa and mNSS neurological function scores. Moreover, compared with the Sham group, there were no significant differences in the protein expression of USP10 and pro-inflammatory cytokines and the number of Iba^+^ microglia in the cerebral cortex of the Sham+VNS group mice. It indicated that VNS exerts no effects on neurological function and neuroinflammation in Sham mice (**Supplementary Figure 1**).

### USP10 was involved in the inhibition of NF-κB signalling pathway by VNS in ischaemic stroke

**Figure 3:**
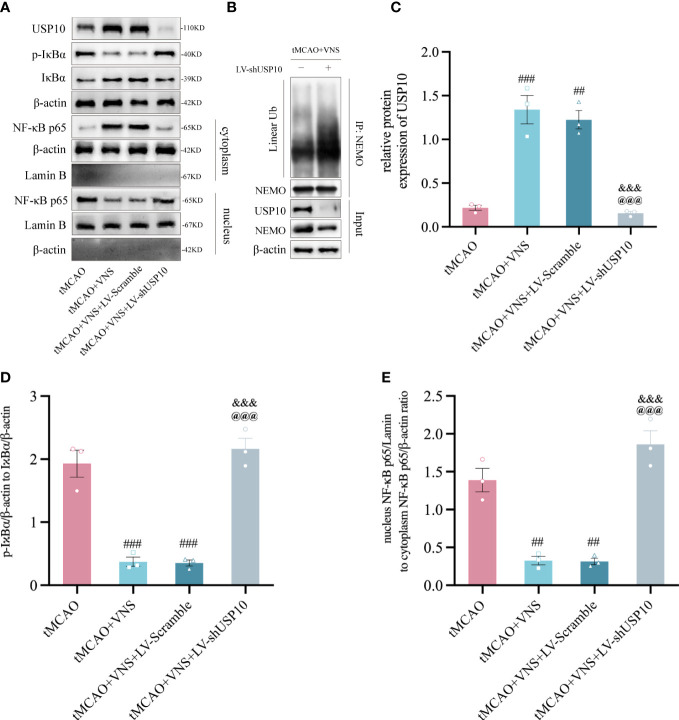
VNS inhibited the NF-κB signaling pathway by elevating the USP10 expression level. **(A)** Western blotting showed the protein expression of USP10, p-IκBα, IκBα, cytoplasmic-p65, and nuclear-p65. β-actin: the control for total or cytoplasmic proteins. Lamin B: the control for nuclear proteins. n=3; **(B)** immunoprecipitation assays showed the effect of USP10 silencing on the linear ubiquitination of NEMO. **(C–E)** Histogram of the USP10 protein expression **(C)**, IκBα phosphorylation ratio **(D)**, and the ratio of nuclear to cytoplasmic NF-κB p65 **(E)**. n=3; Data are mean ± SEM. ###P<0.001, ##P<0.01, and #P<0.05 vs. tMCAO group. @@@P<0.001 vs. tMCAO+VNS group. &&&P<0.001 vs. tMCAO+VNS+LV-Scramble group.

To determine if VNS suppresses the NF-κB signaling pathway by upregulating USP10, the expressions of USP10, IκBα, p-IκBα, nuclear-p65, and cytoplasmic-p65 were assessed in the ischaemic penumbra of cerebral cortex at 72 hr after reperfusion. The nuclear/cytoplasmic NF-κB p65 represents the nuclear translocation of the NF-κB p65 protein, while the p-IκBα/IκBα reflects IκBα’s phosphorylation level. The reduction in phosphorylation of IκBα and nuclear translocation of p65 suggests suppression of the NF-κB signalling pathway and vice versa. We found a significant increase in USP10 expression and a decrease in the ratio of p-IκBα/IκBα and nuclear/cytoplasmic p65 in the tMCAO+VNS group compared with the tMCAO group. However, USP10 expression was significantly suppressed in the MCAO+VNS+LV-shUSP10 group. USP10 expression was significantly suppressed while the ratio of p-IκBα/IκBα and nuclear/cytoplasmic p65 were elevated in the tMCAO+VNS+LV-shUSP10 group compared with tMCAO+VNS group (**Figures 3A–E**). These results indicated silencing of USP10 reverses the inhibitory role of VNS treatment in the NF-κB signaling pathway.

Since the ubiquitination of NEMO is essential for IKK activation which consequently triggers the activation of the NF-κB pathway. To investigate whether the inhibitory effect of VNS on NF-κB is caused by the deubiquitination of NEMO mediated by the upregulation of USP10. We performed immunoprecipitation assay with the tMCAO+VNS+LV-Scramble group and the tMCAO+VNS+LV-shUSP10 group to evaluate the effect of USP10 silencing on the ubiquitination level of NEMO. It showed that USP10 silencing caused an increased level of linear ubiquitination of NEMO (**Figure 3B**). Our results indicated that VNS upregulated USP10 expression and suppressed NF-κB pathway activation, which may be due to the reduction of NEMO ubiquitination by USP10 upregulation.

### USP10 silencing reversed the VNS-induced suppression of microglial activation in mice following ischaemic stroke

#### The morphological alterations of microglia

**Figure 4:**
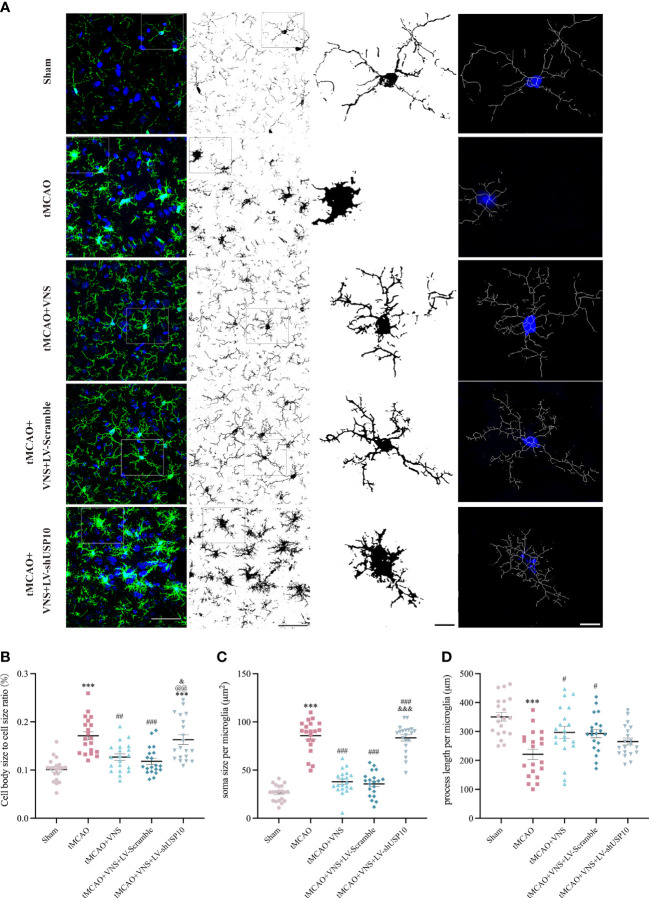
Quantification and illustration of morphological characteristics of microglia following skeleton reconstruction. **(A)** Graphical illustration of soma size cell body size, and process length of microglia for each group. Left column: maximum intensity projection of Z-stack images (blue: nuclei identified with the fluorescent DNA stain DAPI; green: microglia marker Iba-1). n=20; Right three columns: the skeleton reconstruction. Scale bar (Left two columns) =50μm, Scale bar (Right two columns) =10μm. **(B)** The quantification of cell body size to cell size ratio (%). **(C)** The quantification of process length per microglia. **(D)** The quantification of soma size per microglia. Data are mean ± SEM. ***P<0.001 vs. Sham group, ###P<0.001, ##P<0.01, and #P<0.05 vs. tMCAO group. @@P<0.01 vs. tMCAO+VNS group. &&&P<0.001, and &P<0.05 versus tMCAO+VNS+LV-Scramble group.

The response of microglia to injury can be manifested as morphological alteration (33). To reveal the role of USP10 in suppressing microglia activation by VNS, morphological changes of microglia were observed using maximum intensity projection and skeletonization reconstruction of Z-stack images obtained by high magnification confocal microscopy. The cell body to cell size ratio shows a very strong positive correlation with microglial activation and accordingly, as shown in **Figures 4A, B**, the cell body size to cell size ratio was significantly higher in the tMCAO group than in the sham group. Moreover, the ratio was reduced by VNS treatment while it was increased by USP10 silencing. Meanwhile, after skeletonization reconstruction, the morphological features of microglia were also characterized by the quantification of process length and soma size per microglia in this study. Large soma size and short process length indicate microglia are activated, while the small soma size and long process length indicate the microglia are in a homeostatic state (35). As shown in **Figures 4C, D**, soma size per microglia in the sham group was small and process length was long, while the soma size was significantly enlarged and the process length was shortened in the tMCAO group, suggesting that tMCAO induced microglial activation. VNS treatment inhibited the activation of microglia induced by tMCAO, as evidenced by the decreased soma size and increased process length. However, the induction of microglia transformation to a homeostatic state by VNS treatment was suppressed by USP10 silencing.

#### The reaction of microglia

**Figure 5:**
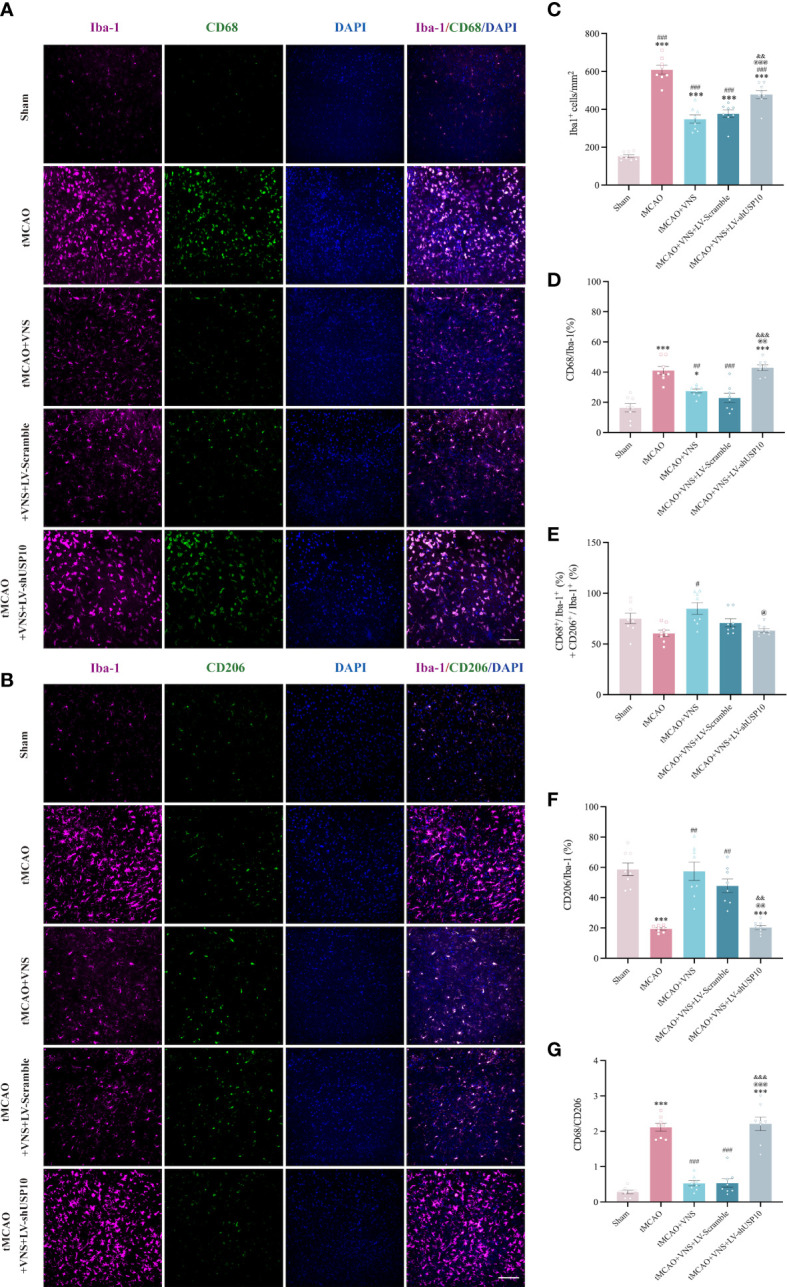
Immunofluorescence shows USP10 silencing reversed the effect of VNS on microglial activation in ischaemic stroke mice. **(A, B)** Representative immunofluorescence images of CD68**(A)** or CD206**(B)** expression in Iba-1^+^ cells. **(C)** Histogram representation of the quantification of Iba-1^+^ cell numbers. **(D)** The ratio of CD68^+^ cells to Iba-1^+^ cells is illustrated as a histogram. **(E)** Quantification of the total reactive microglia by the sum of CD68^+^ + CD206^+^ cell to Iba-1^+^ cell ratio. **(F)** The ratio of CD206^+^ cells to Iba-1^+^ cells is illustrated as a histogram. **(G)** Quantification of CD68^+^/CD206^+^ ratio. n=8, Scale bar =100 μm. Data are mean ± SEM. ***P<0.001 and *P<0.05 vs. Sham group, ###P<0.001, ##P<0.01, and #P<0.05 vs. tMCAO group. @@@P<0.001, @@P<0.01, and @P<0.05 vs. tMCAO+VNS group. &&&P<0.001 and &&P<0.01 vs. tMCAO+VNS+LV-Scramble group.

**Figure 6:**
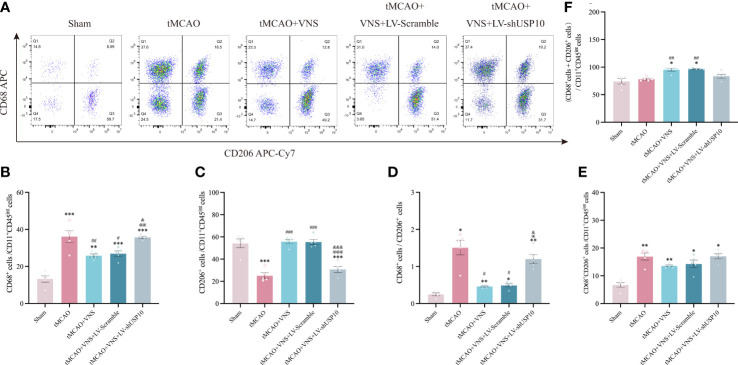
Flow cytometry shows USP10 silencing reversed the effect of VNS on microglial activation. **(A)** Quantification of CD68^+^ and CD206^+^cells after gating strategy sorting of CD11b^+^CD45^int^ microglia. **(B)** The ratio of CD68^+^ cells to CD11b^+^CD45^int^ cells is illustrated as a histogram. **(C)** The ratio of CD206^+^ cells to CD11b^+^CD45^int^ cells is illustrated as a histogram. **(D)** Quantification of CD68^+^/CD206^+^ ratio. **(E)** Quantification of CD68^+^CD206^+^ cells to CD11b^+^CD45^int^ cells ratio. **(F)** Quantification of the total reactive microglia by the sum of CD68^+^ + CD206^+^ cells to CD11b^+^CD45^int^ ratio. n=5. Data are mean ± SEM. ***P<0.001, **P<0.01, and *P<0.05 vs. Sham group, ###P<0.001, ##P<0.01, and #P<0.05 vs. tMCAO group. @@@P<0.001, @@P<0.01 and @P<0.05 vs. tMCAO+VNS group. &&&P<0.001, and &P<0.05 vs. tMCAO+VNS+LV-Scramble group.

Since the detection of CD68 is commonly interpreted as a response to a pro-inflammatory state, while the detection of CD206 is interpreted as a response to an anti-inflammatory state, we further distinguished the response of microglia among the five groups by analyzing the proportion of each type of microglia relative to the total number of Iba-1^+^cells by immunofluorescence. The number of CD68^+^ or CD206^+^ cells after the sort of CD11b^+^CD45^int^ microglia by flow cytometry were also calculated in this study. The results are presented in **Figures 5A–C**. The total number of Iba-1^+^ microglia was significantly increased in the tMCAO group compared to the Sham group. VNS treatment reduced the increased number of microglia induced by tMCAO, while this effect was inhibited by the silencing of USP10. As illustrated in **Figures 5D**, **6A**, the percentage of CD68^+^ microglia were higher in the tMCAO group than in the Sham group. Importantly, the percentage of CD68^+^ microglia was significantly reduced, whereas the percentage of CD206^+^ microglia was significantly increased in the tMCAO+VNS group, in comparison with the tMCAO group (**Figures 5D, F**, **6A–C**). Moreover, the percentage of CD68^+^ microglia was upregulated, while the ratio of CD206^+^ microglia was downregulated in the tMCAO+VNS+LV-shUSP10 group compared to the tMCAO+VNS group. Interestingly, compared to the sham group, the number of CD68+CD206+ cells were elevated in the other four groups (**Figure 6E**). Furthermore, tMCAO+VNS treatment upregulated the total reactive microglia ratio (percentage of CD68^+^ + CD206^+^ microglia), while silencing USP10 dampened this effect (**Figures 5E**, **6F**). Subsequently, to better elucidate the dynamics of microglia pro- or anti-inflammatory reaction, the CD68/CD206 ratio was also measured (**Figures 5G**, **6D**). A ratio higher than 1 indicates that microglia tend to have a pro-inflammatory function. In this study, this ratio was highly increased in the tMCAO group but greatly reduced in the tMCAO+VNS group. Significantly, USP10 silencing blocked the reduction of the CD68/CD206 ratio by VNS treatment.

### USP10 was involved in regulating inflammatory cytokines by VNS in ischaemic stroke mice

**Figure 7:**
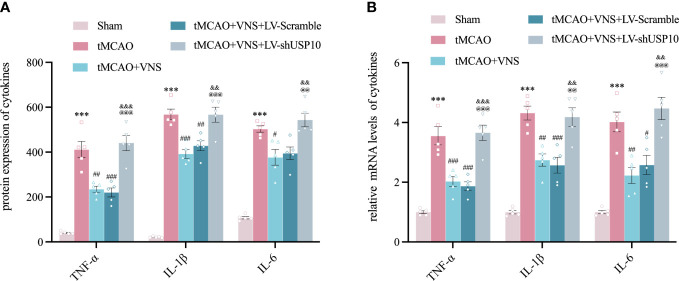
Silencing USP10 reversed the inhibitory role of VNS on inflammatory cytokine expression. **(A, B)** The expression of TNF-α, IL-1β, and IL-6 in the cerebral cortex’s ischaemic penumbra were detected by ELISA (A, n=6) and RT-qPCR (B, n=6). Data are mean ± SEM. ***P<0.001 vs. Sham group, ###P<0.001, ##P<0.01 and #P<0.05 vs. tMCAO group. @@@P<0.001 and @@P<0.01 vs. tMCAO+VNS group. &&&P<0.001, and &&P<0.01 vs. tMCAO+VNS+ LV-Scramble group.

To determine the role of USP10 silencing in VNS-induced inhibition of pro-inflammatory cytokines, the expressions of TNF-α, IL-1β and IL-6 in the ischaemic penumbra of cerebral cortex were detected at 72hr by RT-qPCR and ELISA. The levels of TNF-α, IL-1β, and IL-6 were obviously increased in tMCAO group compared with the Sham group. However, VNS treatment significantly suppressed the upregulation of these cytokines induced by tMCAO. On the contrary, silencing of USP10 expression blocked the reduction of these cytokines resulting from VNS treatment (**Figures 7A, B**).

### USP10 silencing reversed the inhibition of astrocyte activation induced by VNS treatment in mice with ischaemic stroke

**Figure 8:**
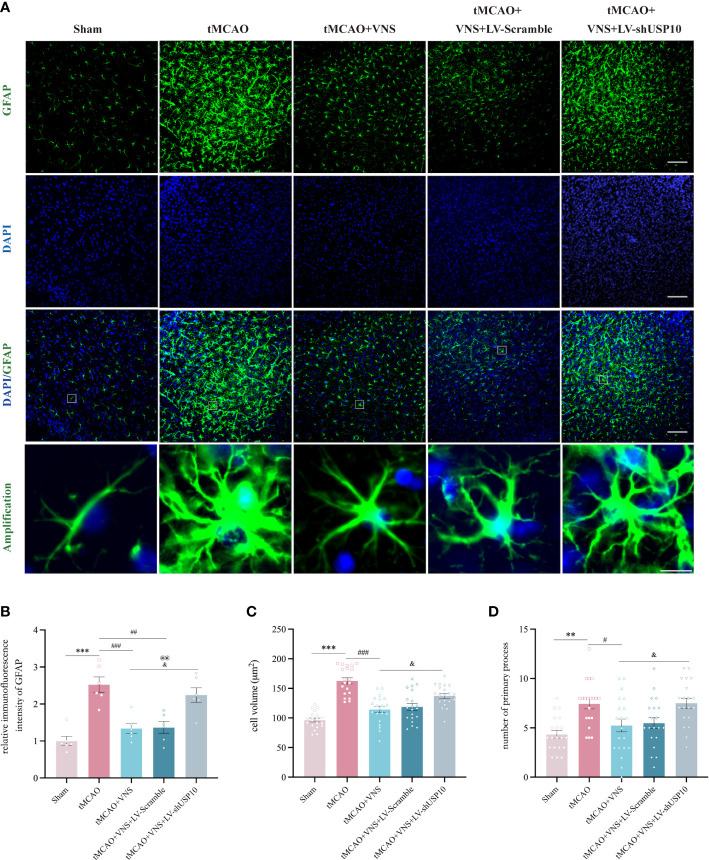
USP10 was involved in the inhibition of astrocyte activation by VNS. **(A)** Immunofluorescence staining of GFAP-positive astrocytes. n=6; **(B)** Quantification of the mean immunofluorescence intensity of GFAP. n=20; **(C)** Quantification of the cell volume. **(D)** Quantification of primary process number. n=20, Scale bar (top three rows) =100 μm; Scale bar (the last row) =10μm. Data are mean ± SEM. ***P<0.001 and **P<0.01 vs. Sham group, ###P<0.001, ##P<0.01, and #P<0.05 vs. tMCAO group. @@P<0.01 vs. tMCAO+VNS group. &P<0.05 vs. tMCAO+VNS+LV-Scramble group.

At 72 hr after tMCAO, GFAP^+^ astrocytes were observed in the ischemic penumbra of the cerebral cortex via immunofluorescence. Astrocyte activation could manifest as increase in cell count, enlargement of cell volume, and extension of processes (36). The mean immunofluorescence intensity of GFAP, the cell volume, and the number of primary processes were quantified (**Figures 8A–D**). The mean immunofluorescence intensity of GFAP (**Figure 8B**), the astrocytes cell volume (**Figure 8C**), and the number of primary processes (**Figure 8D**) were significantly increased in the tMCAO group compared with the sham group. However, these changes were suppressed by VNS treatment in tMCAO+VNS group, which demonstrated that VNS treatment can inhibit the activation of astrocytes. In contrast, USP10 silencing reversed the suppression of astrocytes activation induced by VNS, as evidenced by an increase in the mean immunofluorescence intensity of GFAP (**Figure 8B**), GFAP^+^ cell volume (**Figure 8C**), and the number of primary processes (**Figure 8D**) in the tMCAO+VNS+LV-shUSP10 group compared with the tMCAO+VNS group.

## Discussion

VNS, a well-established neuromodulation method with a long history and promising applications, is approved by the FDA for the treatment of specific neurological and psychiatric disorders, including epilepsy, cluster headache, and depression (37). However, its application for other neurological disorders remains to be clarified (11). Studies have extended the potential treatment of VNS for several neurological conditions regarding limb, swallowing, and cognitive deficits following ischaemic stroke, Alzheimer’s disease, or traumatic cerebral injury (24, 38, 39). Several studies have shown that VNS is a potential treatment for ischaemic stroke (40) which improves motor recovery (41). Similarly, we demonstrated that VNS didn’t affect neurological function without ischaemic stroke. Moreover, our study showed that VNS can reduce infarct volume and alleviate neurological deficits induced by ischaemic stroke.

USP10, a member of the ubiquitin-specific protease family of cysteine proteases, has been demonstrated to serve as an anti-stress factor under a variety of stressful circumstances, including oxidative stress, thermal shock, and viral infection (42). USP10 was shown to decrease the generation of reactive oxygen species (ROS) when exposed to an oxidant, hence preventing ROS-dependent apoptosis (43). Cerebral ischemia triggers the generation of ROS in the brain. Wang et al. found the protective effect of USP10 in the progression of ischaemic stroke by inhibiting inflammation and apoptosis through interaction with transforming growth factor-β activated kinase-1 (TAK1). TAK1 is highly expressed in the brain and controls viability and inflammation through multiple downstream effectors, including the MAP kinases p38 and JNK, and the transcription factor NF-κB (20). Our results demonstrated a gradual decrease of USP10 level following ischaemic stroke, while this level was upregulated by VNS treatment resulting in reduced cerebral infarct volume and alleviated neurological deficits; however, this protective effect was blocked by USP10 silencing. The present study showed that the upregulation of USP10 exerted by VNS treatment was most significant at 72h, a time point that is considered an important time frame for ischaemic stroke therapies (44). Therefore, in order to explore whether USP10 is the potential mediator for vagus nerve stimulation to alleviate neuroinflammation in ischaemic stroke, we chose 72h as the time point for the follow-up experiments. Our previous study also showed that VNS significantly inhibited NLRP3 inflammatory inflammasome and improved neurological deficits at 72h after tMCAO (15).

Recent studies have shown that the protective effects of VNS against ischemic stroke are mediated by the reduction of neuroinflammation (45), decrease in cell autophagy and apoptosis (16, 28), protection of the blood-brain barrier (BBB) (46), angiogenesis (23), as well as suppression of spreading depolarization (45). Neuroinflammation has been demonstrated to play a crucial role in the pathogenesis of stroke (47). The NF-κB signaling pathway, a critical mediator in neuroinflammation, is broadly activated in the brain after ischaemic stroke. Since aberrant activation of NF-kB is associated with a variety of pathogenic events, negative control and correct termination of NF-kB signaling are required to preserve cellular homeostasis. Following ischaemic stroke, VNS can inhibit Toll-like receptor 4/NF-κB expression (16, 48). Long et al. found that the alleviation of dysphagia symptoms by VNS may involve inhibition of inflammatory response, an increase of remyelination, and induction of angiogenesis as a result of reduced levels of NF-κB in the brain white matter of rats models with cerebral ischemia (49). The present study showed that VNS treatment inhibited the activation of the NF-κB signaling pathway as evidenced by the decrease in phosphorylation of IκBα and nuclear translocation of NF-κB p65. In the cytoplasm, IKKγ/NEMO can be further modified by polyubiquitin with linear linkages, which may be required for the optimum activation of IKK (50). USP10 has the capacity to cleave the linear polyubiquitin chains bound to NEMO and destroy the ubiquitin scaffolds essential for IKK activation and subsequent NF-κB activation (5). Upon damage, USP10 suppressed NF-κB activation and the production of inflammatory cytokines (5, 19), while the deletion of USP10 increased the activation of NF-κB (19, 30). Therefore, it is reasonable to speculate that VNS may inhibit the NF-κB signaling pathway and suppress neuroinflammation by upregulating the expression of USP10. We found a significant increase in USP10 expression and a decrease in the phosphorylation ratio of IκBα and the nuclear translocation of p65 protein. However, the USP10 silencing reversed the inhibition of VNS treatment in the NF-κB signaling pathway. Most importantly, we found that USP10 silencing caused an increased level of linear ubiquitination of NEMO. Our results implied that the VNS-induced increase in USP10 level is associated with the deubiquitination of NEMO and finally the inhibition of NF-κB signaling pathway in ischaemic stroke.

Dysregulated immunity, as a prominent feature of neurological disorders, is an attractive treatment target (51). Neuroinflammation involving glial cells serves as a critical contributor to neurological deficits (52). Microglia, among the most important cells participating in neuroinflammation after ischaemic stroke (35), are the unique highly specialized tissue-resident macrophages localized in the parenchyma of the CNS, which constantly monitor the CNS microenvironment and rapidly respond to stimuli by acquiring a reactive phenotype characterized by enlarged cell body and shortened process length (53). Currently, microglia are regarded to have a dual role in neurological recovery, as they can switch to either a harmful pro-inflammatory phenotype or a neuroprotective regulatory phenotype. In the pro-inflammatory phenotype, microglia can produce deleterious inflammatory mediators with surface markers such as CD9, CD10, CD11b, CD16, CD32, CD68, and CD86. In contrast, in the anti-inflammatory phenotype, microglia can express anti-inflammatory molecules such as tumor growth factor-β (TGF-β), interleukin-4 (IL-4), interleukin-10 (IL-10), and surface markers Arg-1, CD163, and CD206 (54). VNS can effectively regulate microglial response to ischaemic stroke (48). Zhao et al. demonstrated that VNS could inhibit IL-17A expression to promote microglia anti-inflammatory reaction and neuroprotection (55). Zhang et al. showed that VNS can promote polarization of microglia from pro- to anti-inflammatory response to alleviate cerebral injury by inhibiting TLR4/MyD88/NF-κB pathway in the acute stage of stroke (16). Our result showed VNS exerts no impact on Iba^+^ microglia in mice without ischaemic stroke. Furthermore, as anticipated, our study established that VNS treatment could reduce the transformation of microglia to pro-inflammatory reaction (CD68^+^Iba^+^) and promote its stabilization in anti-inflammatory reaction (CD206^+^Iba^+^) after ischaemic stroke, similar to the findings in other studies (56). More importantly, this effect of VNS on microglia response was achieved by upregulating USP10 and thus inhibiting the NF-κB signaling pathway from increasing the number of anti-inflammatory microglia. These results indicated that VNS could upregulate USP10 and subsequently inhibit NF-κB signaling pathway, thus preventing microglia activation.

In a pro-inflammatory state following ischaemic stroke, activated microglia can produce a variety of pro-inflammatory cytokines such as TNF, IL-1β, and IL-6 (57). These cytokines can affect CNS functions and disrupt the BBB (58). In a study of a rat model with ischaemic stroke, VNS could attenuate BBB destruction by reducing injuries to endothelial cells, astrocytes, and tight junctions in the neurovascular unit (46). As noted in the present study, VNS treatment partly blocked the increased levels of TNF-α, IL-1β, and IL-6 in the cerebral penumbra induced by the activation of the NF-κB signaling pathway after tMCAO. However, this protective role of VNS was suppressed by USP10 silencing, which further indicated that USP10 plays a critical role in the anti-neuroinflammation effect of VNS against ischaemic stroke.

Pro-inflammatory microglia can provoke the transformation of resting astrocytes to reactive astrocytes called reactive astrogliosis, thus inducing inflammation and neurotoxicity on the one hand. The inflammatory response of reactive astrocytes could further exacerbate neuroinflammation and release detrimental mediators to exert neurological injury (59), thus hindering neurological recovery on the other hand (60). Therefore, inhibition of excessive astrogliosis after ischaemic stroke is beneficial (61). Accordingly, one study have demonstrated that VNS inhibits astrocyte activation induced by cerebral ischaemia (62). In the present study, USP10 was required for VNS to attenuate neuroinflammation and microglial activation, we also evaluated whether silencing USP10 affects astrogliosis and our results demonstrated that the inhibition of astrocyte activation by VNS was significantly dampened after USP10 silencing.

## Conclusion

In summary, this study provides clear evidence that VNS attenuates the neuroinflammation induced by ischaemic stroke. Our results revealed that enhanced USP10 was required for VNS to attenuate neurological deficits, neuroinflammation, and glial cell activation in ischaemic stroke by regulating NF-κB signaling pathway.

Taken together, these evidences suggest that increasing USP10 expression in the brain is a preventative strategy for ischaemic stroke-induced neuroinflammation response and subsequent brain damage. In conclusion, VNS is a neuromodulation therapy for multiple neurological disorders, however, the mechanism by which VNS affects the CNS has not yet been well elucidated, limiting the therapeutic optimization and requiring therefore further exploration.

## Data availability statement

The original contributions presented in the study are included in the article/**Supplementary Material**. Further inquiries can be directed to the corresponding author.

## Ethics statement

The animal study was reviewed and approved by Animal experimental ethical inspection of the Second Affiliated Hospital of Chongqing Medical University.

## Author contributions

CX, XG, and CL designed the experiments and supervised the work. CX, XG, GL and HT performed the experiments. CX and XG analyzed the data. CX, GL, and HT prepared the figures. CX and CL wrote and revised the manuscript. All authors read and approved the final manuscript. All authors contributed to the article and approved the submitted version.

## Supplementary material

Supplemental Figure 1 The effect of VNS on USP10 expression and neuroinflammation in the cerebral cortex of mice without ischaemic stroke (Sham+VNS). **(A)** The USP10 protein expression on 1d and 3d after sham surgery and VNS treatment were assessed by western blot. **(B)** Histogram of the USP10 protein expression. n=3. **(C)** The expression of TNF-α, IL-1β, and IL-6 were detected by ELISA. n=5. **(D)** Immunofluorescence images of Iba-1^+^ cells. Scale bar =100μm. n=5. **(E)** Histogram representation of the quantification of Iba-1+ cell numbers. n=5. ns, no significance.
