# Supplementary figures and images for: USP10 is a potential mediator for vagus nerve stimulation to alleviate neuroinflammation in ischaemic stroke by inhibiting NF-κB signalling pathway
Source: Front Immunol. 2023 Apr 20;14:1130697. doi: 10.3389/fimmu.2023.1130697 (PMC10157167; doi:10.3389/fimmu.2023.1130697)

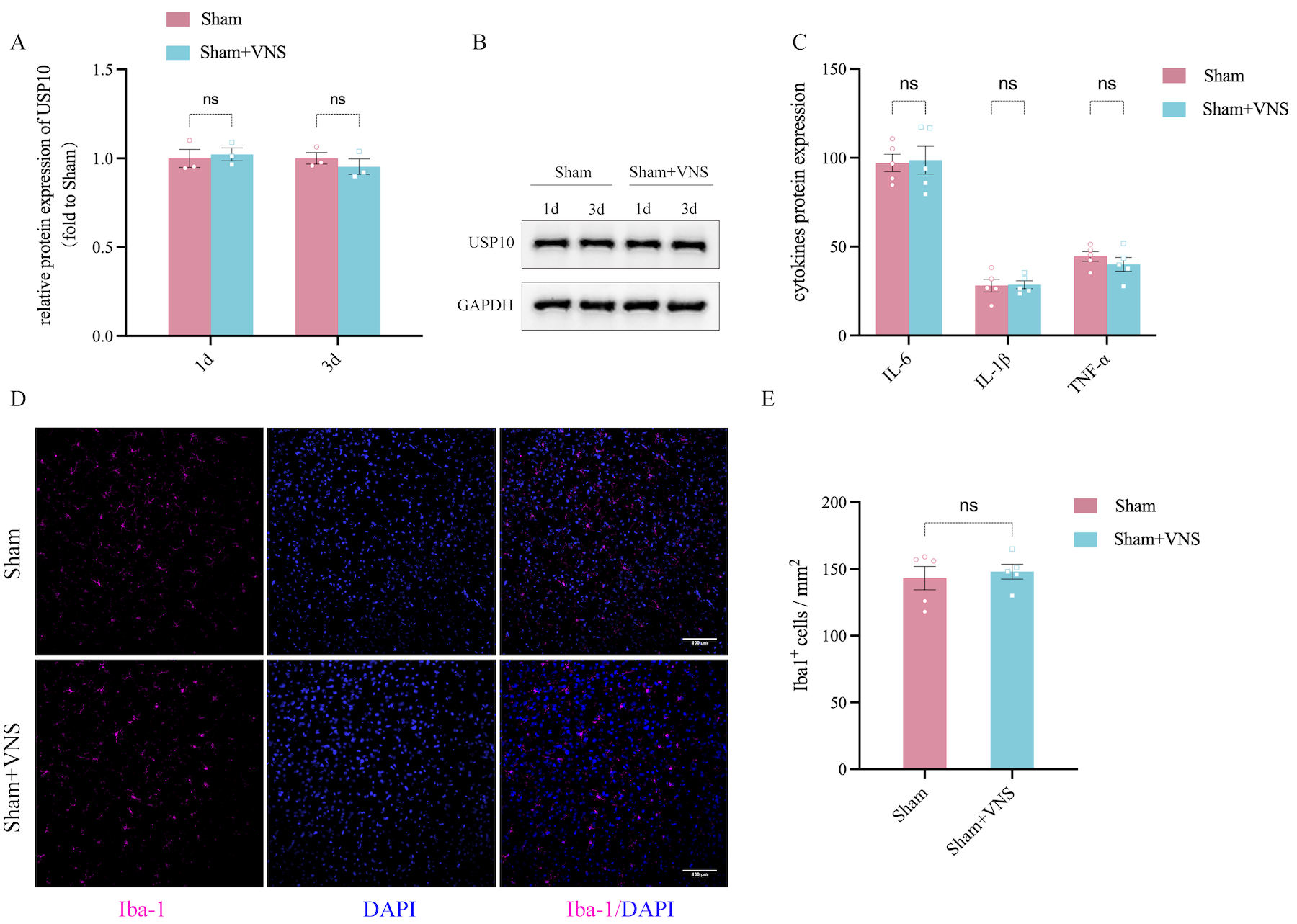

Supplement: Supplemental Figure 1 — The effect of VNS on USP10 expression and neuroinflammation in the cerebral cortex of mice without ischaemic stroke (Sham+VNS). (A) The USP10 protein expression on 1d and 3d after sham surgery and VNS treatment were assessed by western blot. (B) Histogram of the USP10 protein expression. n=3. (C) The expression of TNF-α, IL-1β, and IL-6 were detected by ELISA. n=5. (D) Immunofluorescence images of Iba-1+ cells. Scale bar =100μm. n=5. (E) Histogram representation of the quantification of Iba-1+ cell numbers. n=5. ns, no significance. [file Image_1.tif]
